# Micro-interventional pre-treatment for nucleus disassembly in the setting of non-cavitating sonic lensectomy: real-world evidence study in 512 cases

**DOI:** 10.1186/s12886-025-04150-4

**Published:** 2025-07-01

**Authors:** Sean Ianchulev, Elizabeth Yeu, Edward H. Hu, Gautam Kamthan, Seth Pantanelli, Paul Singh, Farrell Tyson

**Affiliations:** 1 Ophthalmology, New York Eye and Ear Infirmary of Mount Sinai, New York, NY USA; 2 Refractive Surgery, Virginia Eye Consultants, Norfolk, VA USA; 3 Wolfe Eye Clinic, Marshalltown, IA USA; 4 Department of Ophthalmology, Penn State College of Medicine, Hershey, PA USA; 5 The Eye Centers of Racine and Kenosha, Racine, WI USA; 6 The Cape Coral Eye Center (Tyson), Cape Coral, FL USA

**Keywords:** miCOR, miLOOP, Femtosecond, Cataract surgery

## Abstract

**Objectives:**

To investigate the effect of adjunct micro-interventional pre-treatment for nucleus disassembly on the surgical efficiency of non-cavitating lensectomy during cataract surgery.

**Methods and analysis:**

12 surgeons performed 512 consecutive cataract extractions using a sonic cavitation-free lensectomy with or without adjunct pre-treatment for nucleus disassembly. There were 2 interventional arms including (1) lensectomy without adjunct pre-treatment and (2) lensectomy with micro-interventional miLOOP pre-treatment.

**Results:**

Successful lensectomy was achieved in all eyes using cavitation-free sonic lensectomy. Average baseline cataract density was 2.28 and 2.39 in the three groups, respectively. Compared to no pre-treatment, nucleus evacuation time was 24% (p = < 0.001) faster with micro-interventional nucleus disassembly. Irrigation/aspiration (I/A) time was 14% faster with the micro-interventional pre-treatment (p = < 0.001). Irrigation fluid use was 24% less with micro-interventional. There was a low rate of capsular tear of 1 case across 512 cases with no other unanticipated complications.

**Conclusion:**

Micro-interventional pre-treatment for nucleus disassembly was associated with improved lensectomy time and fluidic efficiency compared to no pre-treatment. Non-cavitating lensectomy with the miCOR lens pen achieved effective fragmentation and extraction in all grades of cataract.

## Introduction

Cataracts are a leading cause of curable vision loss worldwide, impacting over 95 million people and contributing to 33% of global blindness [1]. Cataract surgery, one of the most frequently performed procedures globally, accounts for over 3.7 million cases annually in the United States alone [2]. Traditional cataract disassembly techniques, such as divide-and-conquer or various chopping methods, typically require the use of ultrasonic energy from the phacoemulsification probe along with assistance from a second instrument [3, 4, 5]. The amount of phacoemulsification energy needed varies depending on the technique and cataract severity, with higher-grade cataracts requiring more energy [6]. This adds to the total energy and heat of ultrasonic cataract removal. Often, multiple attempts are required to initiate and propagate nucleus fracture, as current chopping methods primarily engage the nucleus complex anteriorly or peripherally and lack instrumentation for posterior access to the nuclear surface.

In an attempt to reduce the dispersive energy during phacoemulsification and protect the endothelium, new adjunct techniques have emerged for the pre-treatment and nucleus disassembly prior to lensectomy and segment removal [7, 8, 9, 10, 11, 12, 13, 14]. Femtosecond laser assisted fragmentation is one such technique where laser energy is used to segment the nucleus prior to initiation of phaco-emulsification [15, 16, 17]. The clinical benefits of femtosecond pre-treatment include reduced ultrasonic energy (substituted for laser energy) and lower cumulative dissipated energy [18, 19, 20, 21]. Sometimes these benefits come at the cost of increased irrigation fluidics, surgical time and reduced cost-effectiveness [22, 23, 24, 25].

**Fig. 1:**
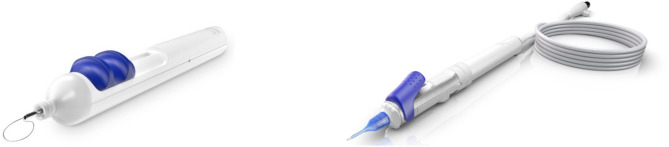
miLOOP Segmentation Device and miCOR Sonic Lens Pen

Another approach for nucleus pre-treatment is micro-interventional mechanical disassembly with the miLOOP device which uses a thin, super-elastic memory shaped nitinol filament to centripetally segment the nucleus (Fig. 1). With mechanical methods no dispersive energy or heat is used. Instead, mechanical force is applied to achieve full-thickness endocapsular centripetal segmentation in addition to cortical mobilization to facilitate I/A [26–29].

The objective of this study was to evaluate the effect of adjunct micro-interventional pre-treatment and nucleus disassembly prior to sonic lensectomy with the miCOR lens pen. Unlike conventional phaco systems, the sonic lensectomy approach is uniquely suited to evaluate adjunct disassembly techniques as it does not emit any dispersive ultrasonic energy in the eye and provides minimal energy cataract extraction conditions [30].

## Methods

### Ethical oversight

We report the clinical experience, surgical outcomes and postoperative results of a consecutive case series of patients with visually significant cataracts who underwent cataract surgery with the sonic lens pen system by 12 surgeons. This study met the criteria under 42 CFR 11.22(b) and was not required to be listed on ClinicalTrials.gov. This retrospective chart review was IRB-approved by WCG IRB (a fully accredited IRB Board) (WCG-20233809). The surgical case logs of 512 consecutive cases which were collected under full patient confidentiality and data were subsequently anonymized. Informed consent was not required to participate in the study. All research adhered to the tenets of the Declaration of Helsinki.

### Patient and public involvement

Patients and the public were not involved in the design, or conduct, or reporting, or dissemination plans of our research.

### Equipment

The miLOOP is a single-use handheld surgical device with a micro-thin filament nitinol loop with shape memory and super elastic properties. The loop can contract from 10.5 mm to 1.5 mm while remaining intact. With a 300 μm diameter, the nitinol filament is designed for elasticity, memory, and cutting force. It allows for nucleus disassembly through a unique, zero-energy centripetal approach, distinct from conventional methods. This process achieves full-thickness, micro-segmentation and disassembly regardless of cataract grade, through a single 1.5 mm incision. It can be controlled using an ophthalmic viscosurgical device, avoiding the need for a phacoemulsification probe and I/A, thus protecting the endothelium during surgery.

The miCOR lens pen is an FDA-cleared handheld fragmentation and lens extraction device (Fig. 1) for cavitation-free lensectomy, which is distinctly different from conventional phacoemulsification systems. Some of the unique properties of the miCOR lensectomy system are summarized in Table 1.

**Table 1:** Sonic Lens pen vs. Standard phacoemulsification system

	Sonic Lens Pen	Ultrasonic Phaco System
Incision	Clear cornea Sub 2.3 mm	Clear cornea Sub 2.3 mm
Tip vibration	Sonic	Ultrasonic
Heat generation inside the eye	None Isothermic	Yes Up to 70℃ [31]
Cavitation energy pulse	None	Yes
Need for high-flow cooling fluidics	None	Yes
IA and Vitrectomy Modules available	Yes	Yes
Hardware	Minimal Handheld probe only	Large Console/Probe
Foot pedal required	No	Yes
Surgical console/ box needed	No	Yes
Sterilization of probe needed	No	Yes

### Study design

This was a non-randomized retrospective consecutive case series of 512 eyes of 496 patients who underwent cataract surgery with the miCOR device with or without pre-fragmentation using the miLOOP. Patients aged over 18 years of age with visually significant cataracts grade 1–4 + by the surgeons pre-operatively were included. All intraocular lens (IOL) power calculations used standard-of-care pre-operative biometry IOL power calculation formulas.

Patients were divided into two interventional groups receiving: no pre-treatment (control group) or micro-interventional pre-treatment (miLOOP). Surgical times of each procedure were recorded, taking note of lens extraction time and cortex removal time. A technician in the operating room recorded lens extraction, cortex removal, and I/A times using a digital timer. Lensectomy time was recorded from entry of the lens pen into the eye until its removal from the eye. Similarly, I/A times were recorded for entry and removal from the eye for cortex and viscoelastic removal. Fluid volumes were recorded for lensectomy and I/A for cortex and viscoelastic removal. Markings on the irrigation fluid bag of the sonic lens pen facilitated measurement of fluid consumption at each step at increments of up to 5mL. Cataracts were graded preoperatively at slit lamp exam referencing the Lens Opacities Classification System III grading system and confirmed intraoperatively by the surgeons under a surgical microscope on a scale from 1 to 4 of increasing density [32].

Surgeons were all experienced phaco surgeons. Additional experience with both the miLOOP and miCOR technologies was required.

### Statistics

Welch’s two-sample t-tests were used to compare continuous variables after assessing data distribution normality through visual inspection of histogram plots. Statistical significance was defined as *p* < 0.05. Statistical and graphing software included Microsoft Excel (Microsoft, Redmond, Washington, USA) and Rstudio (Version 2023.09 + 463, Posit Software, Boston, MA).

## Results

A total of 512 consecutive eyes underwent cataract surgery for visually significant cataracts using the miCOR (Table 2). Twelve surgeons at multiple sites in the US performed the procedures. The majority of eyes underwent micro-interventional pre-treatment (miLOOP) followed by sonic miCOR lensectomy. The mean grade of cataract density was similar across groups (*p* = 0.14).

**Table 2:** Results of the Control versus Pre-treatment groups

	No Pre-treatment^a^	miLOOP Pre-treatment^b^	Total
**N**	163	349	512
**Lens Grade Average**	2.28	2.39	
**Lens Grade SD**	0.65	0.67	
***P***-value*			0.08
**N**	161	349	510
**Lensectomy Time Average (seconds)**	143.46	109.34	
**Lensectomy Time SD**	87.94	92.32	
***P***-value*			**< 0.001**
**N**	153	340	493
**Nucleus Removal Fluid Average (mL)**	24.58	24.77	
**Nucleus Removal Fluid SD**	9.34	9.23	
***P***-value*			0.86
**N**	151	337	488
**IA Time Average (seconds)**	14.58	12.56	
**IA Time SD**	7.24	7.50	
***P***-value*			**< 0.001**
**N**	157	343	500
**IA Fluid Average (mL)**	59.89	44.66	
**IA Fluid SD**	33.35	33.20	
***P***-value*			**0.007**

^a^ -ML = no pretreatment group; ^b^ +ML = miLOOP pretreatment group; * *p* < 0.05 deemed significant as indicated (bold)

The micro-interventonal pre-treatment group exhibited a significantly faster lensectomy time compared to the group which received no pre-fragmentation (*p* < 0.001). I/A time was faster with micro-interventional pre-treatment than no pre-treatment (*p* < 0.001). Using miLOOP resulted in the faster procedure over both groups. Surgeons varied in number of micro-interventional cuts made (from 1 to 4 cuts), with grade 1 lenses averaging 1.88 (standard deviation, SD 0.49) cuts, grade 2 lenses averaging 2.22 (SD 0.76) cuts, grade 3 lenses averaging 1.98 (SD 0.61) cuts, and grade 4 lenses averaging 1.83 (SD 0.38) cuts.

The mean fluid consumption during nucleus extraction was lower for the micro-interventional group (*p* < 0.001) than the control group (*p* < 0.001) during I/A by approximately 15mL.

There was 1 case of intraoperative posterior capsular tear in the micro-interventional group; otherwise, there were no unanticipated complications. 512 cases were performed but not all data was captured for each step and each case, accounting for the different N in each category.

## Discussion

The results of our study demonstrate that micro-interventional pre-treatment is associated with faster speed of nucleus extraction and faster I/A time compared to no pre-treatment in the setting of sonic lensectomy. The study also quantified the surgical performance of a first-of-a-kind handheld sonic lensectomy system which can achieve non-cavitating fragmentation and extraction of the nuclear and cortical segments of the cataract using no dispersive energy or heat. The non-emulsifying lens pen showed few complications and successfully completed all-grade cataract extractions. There was a very low rate of posterior capsular events which is comparable to what is typically seen with conventional phacoemulsification, [32] as well as previously published data with the miCOR [30].

This study explores potential strategies to enhance surgical efficiency and reduce operational costs in cataract surgery by leveraging advanced technologies, novel interventional techniques, and optimized intraoperative workflows. Our findings demonstrate that micro-interventional pre-treatment can decrease nucleus extraction time by approximately 24%, regardless of cataract severity, thereby improving overall surgical efficiency. Additionally, the miCOR device’s handheld design, along with its disposable tip, eliminates the need for the sterilization of phacoemulsification handpieces—a process that can take up to 30 min per probe in high-volume surgical settings. The disposable nature also frees staff time and resources spent on sterilization and repairs for damaged equipment. These features not only simplify surgical preparation but also contribute to cost reductions, offering advantages beyond the initial investment in phacoemulsification equipment.

This real-world evidence study has some inherent limitations. Real-world studies are non-randomized and unmasked which makes them prone to inherent selection and observation bias. These factors, combined with differences in practice patterns among the surgeons, equipment availability, and patient populations, contributed to the uneven and imbalanced distribution across the two treatment groups, with the majority of patients in the micro-interventional miLOOP arm. It was noted, however, that the baseline characteristics such as cataract grade are relatively balanced between groups with slightly more severe, harder cataracts in the micro-interventional group. Confirmation of cataract grade intraoperatively under a surgical microscope is a potential limitation of the study as it differs from the accepted approaches conducted at the slit lamp, possibly enabling heterogeneity in grading. Another limitation of the study is that only surgical performance and intraoperative outcomes were collected, not pre- or post-operatively. Post-operative outcomes such as corneal edema and visual recovery are certainly important and should be evaluated in future studies. Furthermore, while we intentionally studied the effect of adjunct pre-treatment for nucleus disassembly in the setting of minimal energy sonic lensectomy, the same findings may or may not carry over to conventional phacoemulsification lensectomy. This will have to be confirmed in a separate study. Also, the 12 surgeons in our study have experience in miLOOP and miCOR micro-interventional techniques. This may impart some selection bias for ophthalmologists who may not be representative of all cataract surgeons. Lastly, discrepancies in data collection resulted in varying sample sizes for each measured intraoperative variable, which could have introduced bias into the analysis.

Our findings further demonstrate that all three surgical approaches with or without adjunct micro-interventional pre-treatment of sonic lensectomy are viable, safe and effective for cataract surgery in a large cohort of consecutive cases. This is important as it provides surgeons with multiple options for successful intervention depending on the healthcare setting and local socio-economic environment.

## Data Availability

The data that support the findings of this study are available from the authors upon reasonable request.
